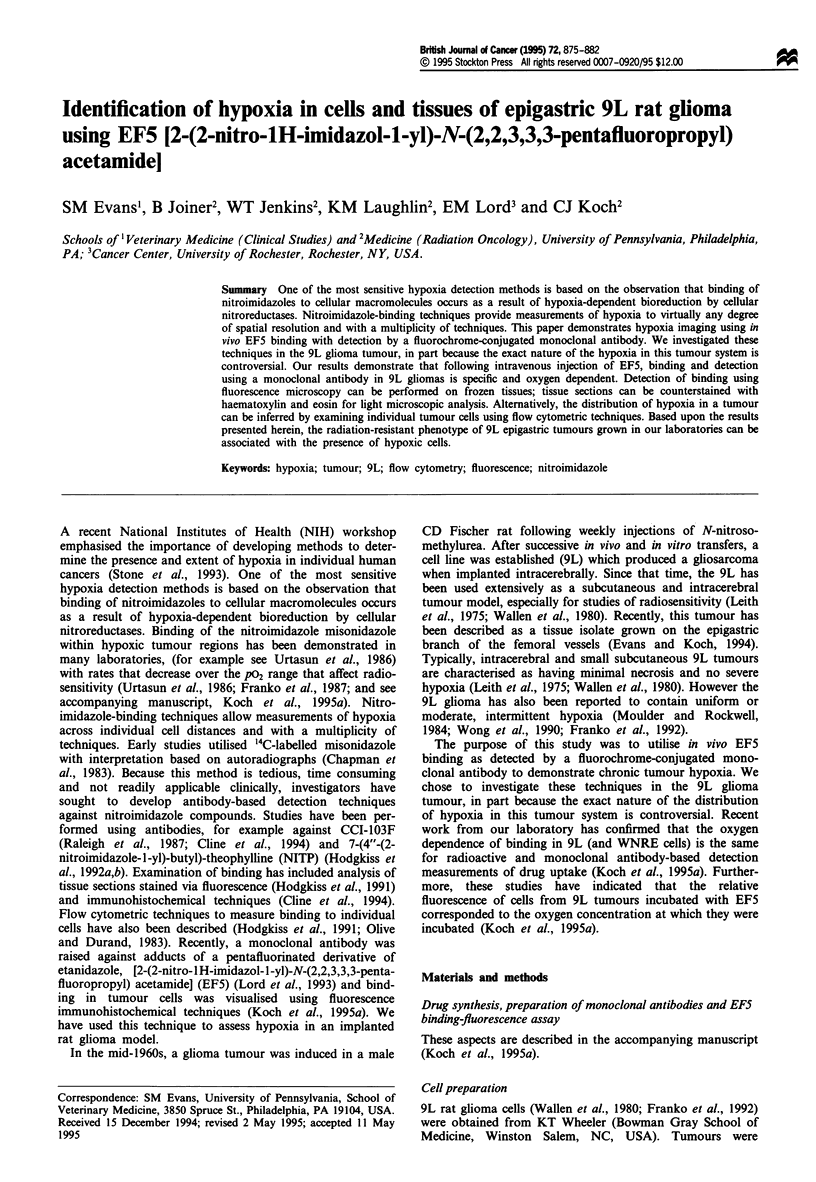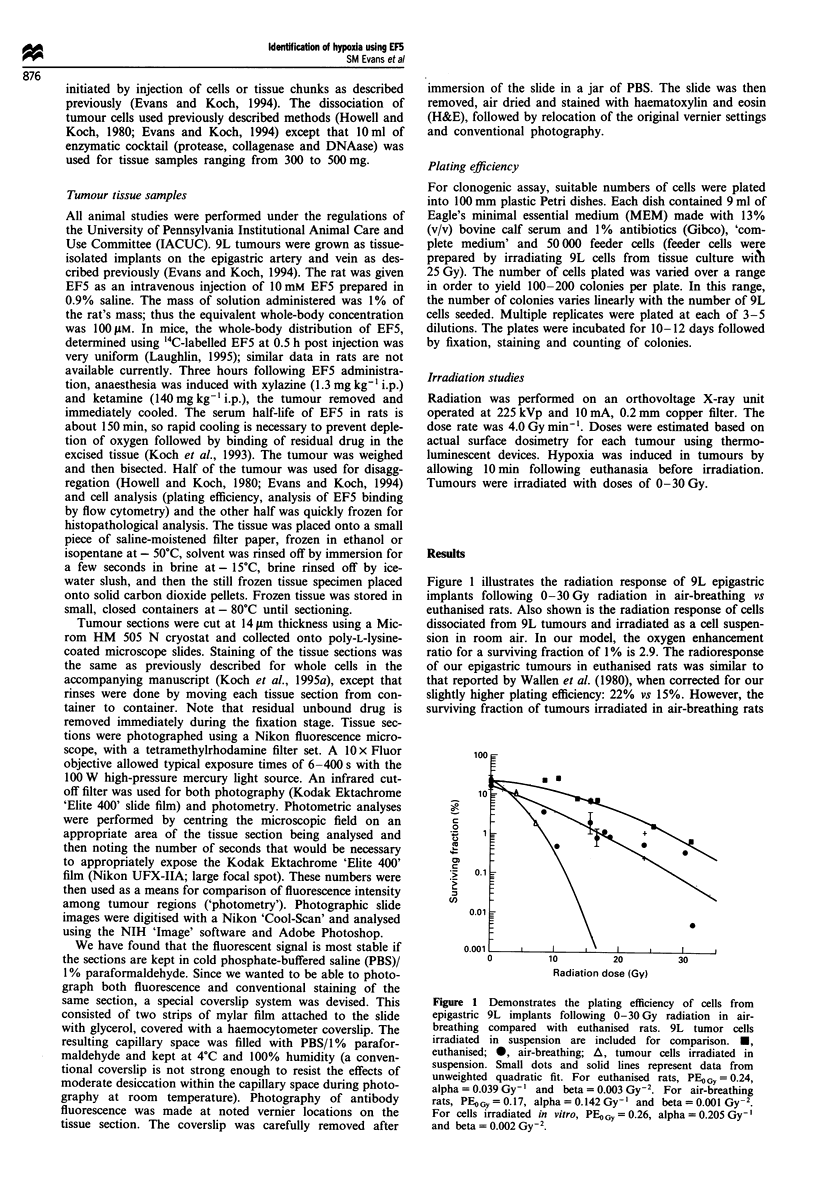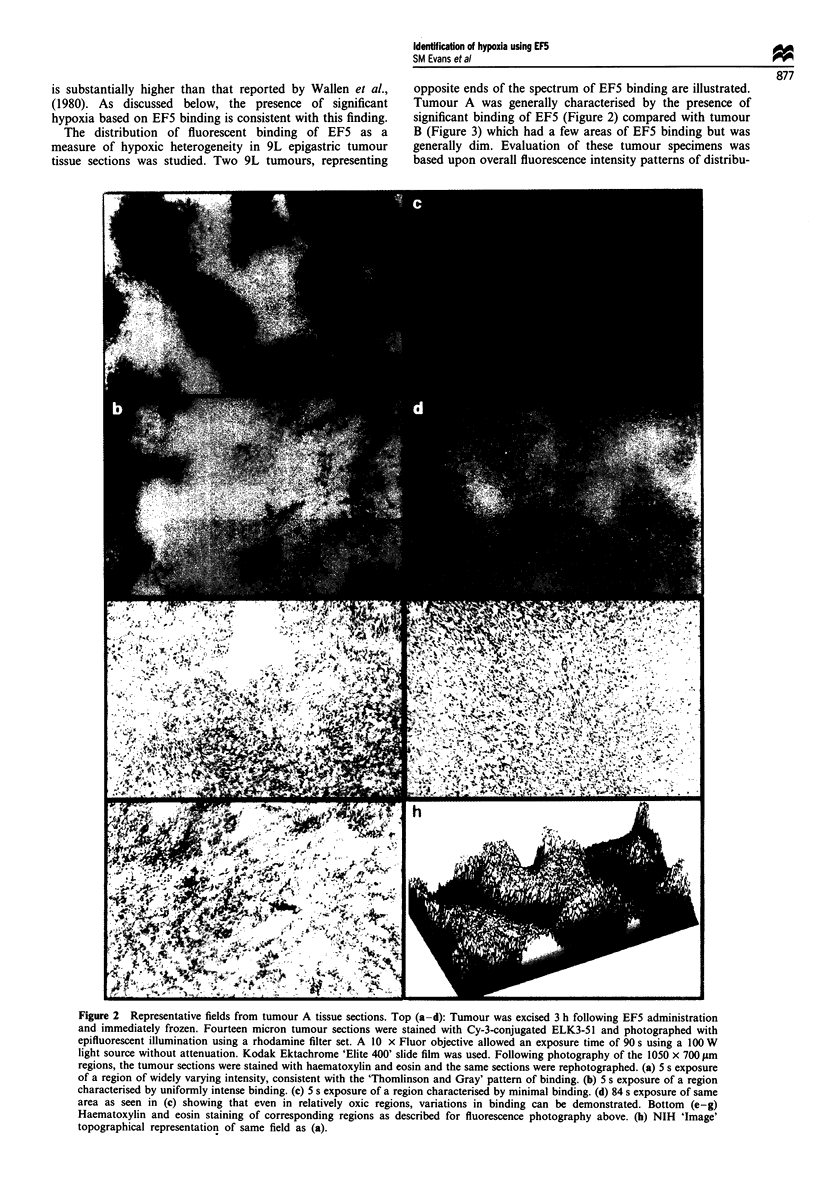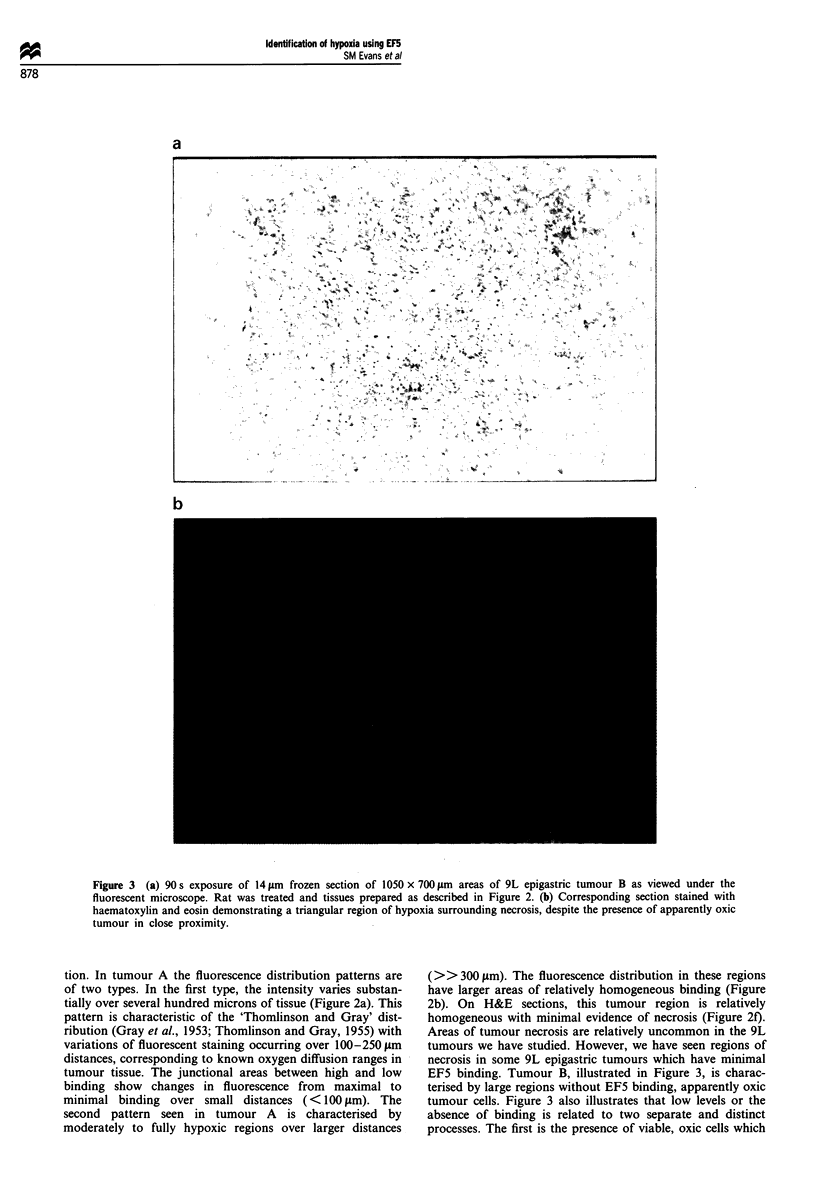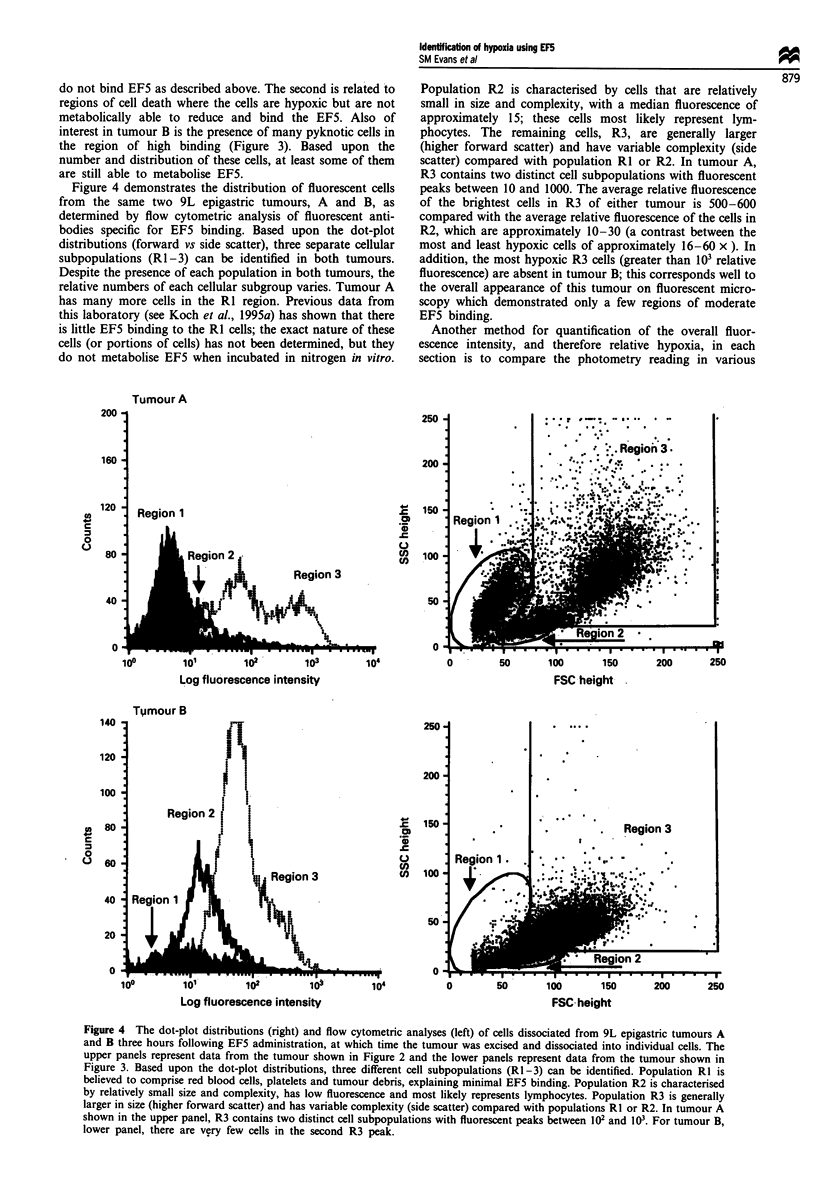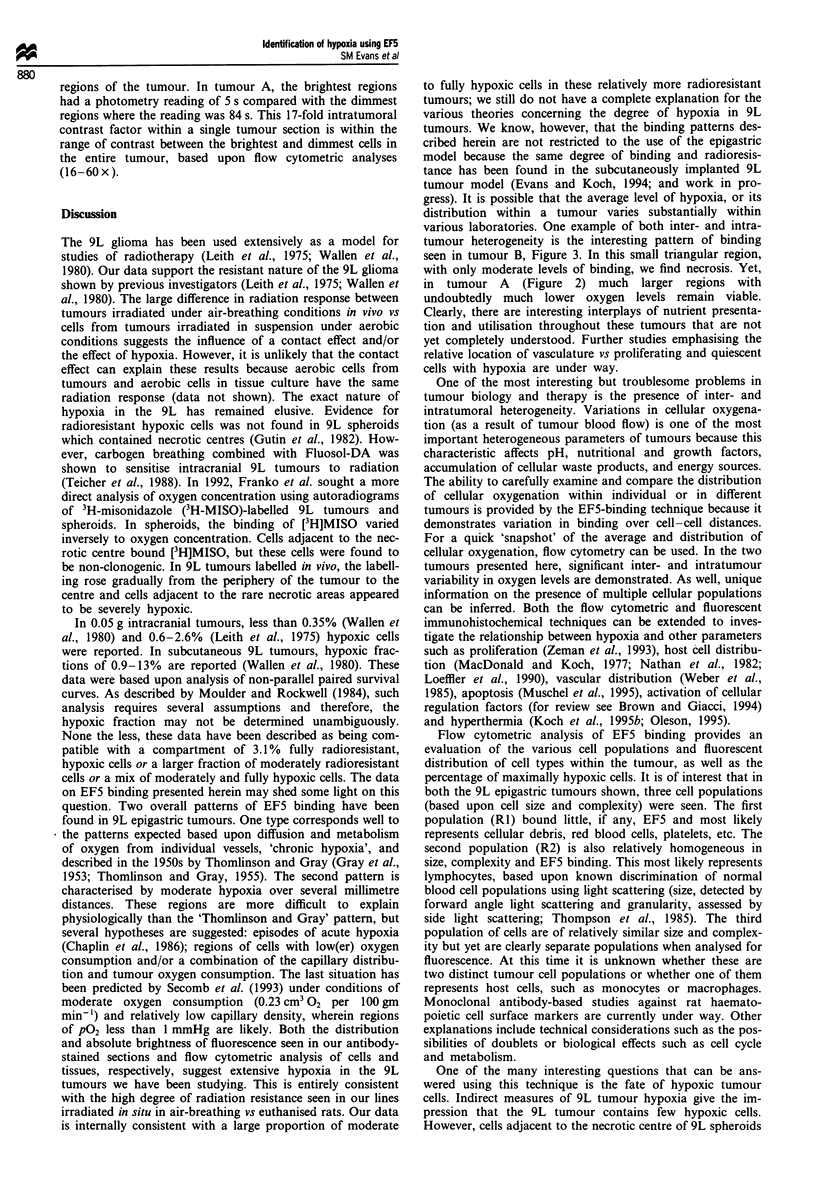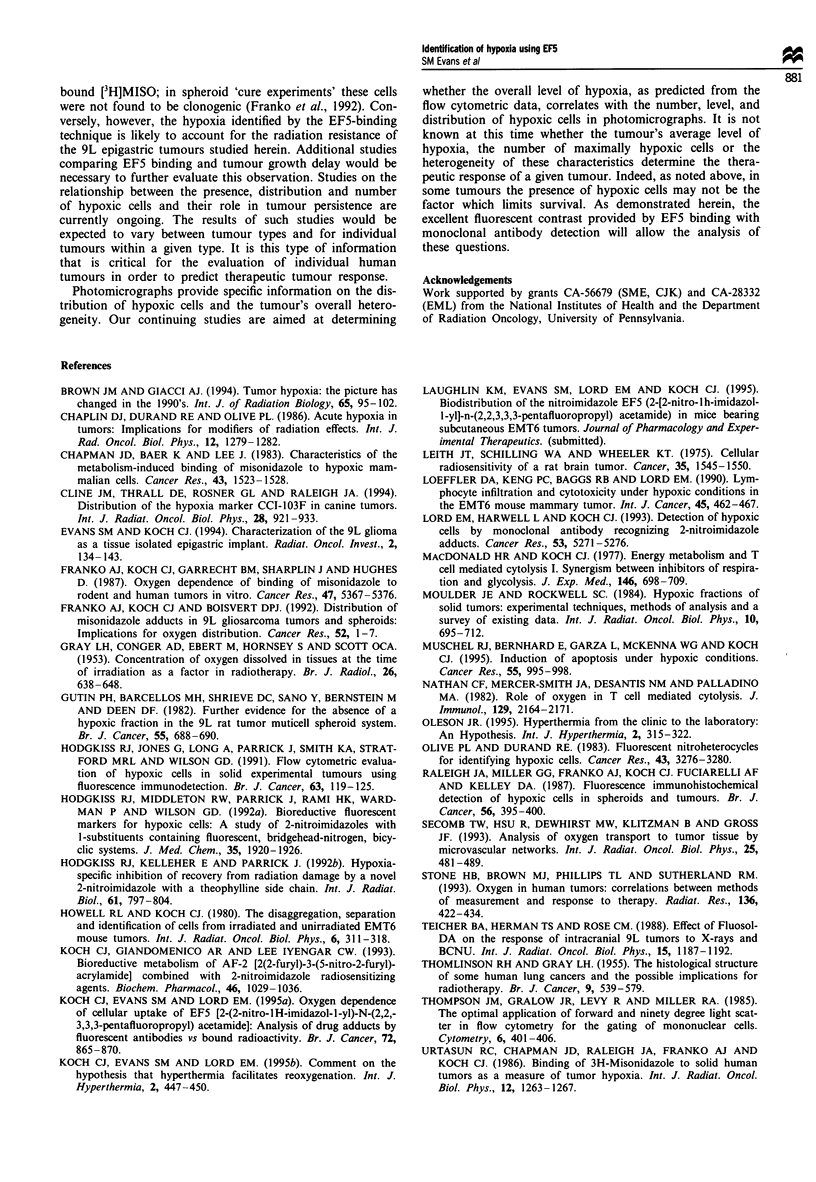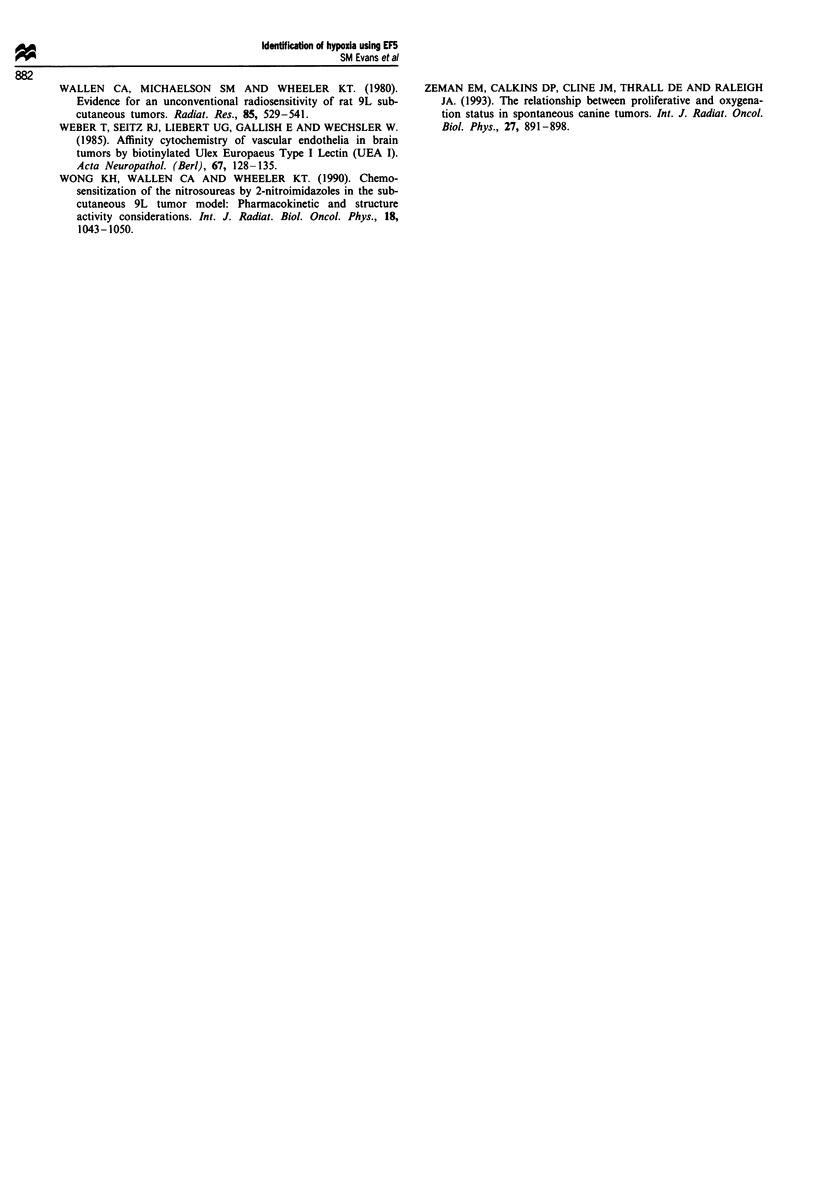# Identification of hypoxia in cells and tissues of epigastric 9L rat glioma using EF5 [2-(2-nitro-1H-imidazol-1-yl)-N-(2,2,3,3,3-pentafluoropropyl) acetamide].

**DOI:** 10.1038/bjc.1995.427

**Published:** 1995-10

**Authors:** S. M. Evans, B. Joiner, W. T. Jenkins, K. M. Laughlin, E. M. Lord, C. J. Koch

**Affiliations:** School of Veterinary Medicine (Clinical Studies), University of Pennsylvania, Philadelphia 19104, USA.

## Abstract

**Images:**


British Journal of Cancer (1995) 72, 875-882
© 1995 Stockton Press All rights reserved 0007-0920/95 $12.00

### Identification of hypoxia in cells and tissues of epigastric 9L rat glioma using EF5 [2-(2-nitro-1H-imidazol-1-yl)-N-(2,2,3,3,3-pentafluoropropyl) acetamide]

SM Evans[1], B Joiner[2], WT Jenkins[2], KM Laughlin[2], EM Lord[3] and CJ Koch[2]

*Schools of [1]Veterinary Medicine (Clinical Studies) and [2]Medicine (Radiation Oncology), University of Pennsylvania, Philadelphia, PA; [3]Cancer Center, University of Rochester, Rochester, NY, USA.*

**Summary** One of the most sensitive hypoxia detection methods is based on the observation that binding of nitroimidazoles to cellular macromolecules occurs as a result of hypoxia-dependent bioreduction by cellular nitroreductases. Nitroimidazole-binding techniques provide measurements of hypoxia to virtually any degree of spatial resolution and with a multiplicity of techniques. This paper demonstrates hypoxia imaging using *in vivo* EF5 binding with detection by a fluorochrome-conjugated monoclonal antibody. We investigated these techniques in the 9L glioma tumour, in part because the exact nature of the hypoxia in this tumour system is controversial. Our results demonstrate that following intravenous injection of EF5, binding and detection using a monoclonal antibody in 9L gliomas is specific and oxygen dependent. Detection of binding using fluorescence microscopy can be performed on frozen tissues; tissue sections can be counterstained with haematoxylin and eosin for light microscopic analysis. Alternatively, the distribution of hypoxia in a tumour can be inferred by examining individual tumour cells using flow cytometric techniques. Based upon the results presented herein, the radiation-resistant phenotype of 9L epigastric tumours grown in our laboratories can be associated with the presence of hypoxic cells.

**Keywords:** hypoxia; tumour; 9L; flow cytometry; fluorescence; nitroimidazole

A recent National Institutes of Health (NIH) workshop emphasised the importance of developing methods to determine the presence and extent of hypoxia in individual human cancers (Stone *et al.*, 1993). One of the most sensitive hypoxia detection methods is based on the observation that binding of nitroimidazoles to cellular macromolecules occurs as a result of hypoxia-dependent bioreduction by cellular nitroreductases. Binding of the nitroimidazole misonidazole within hypoxic tumour regions has been demonstrated in many laboratories, (for example see Urtasun *et al.*, 1986) with rates that decrease over the $pO_2$ range that affect radiosensitivity (Urtasun *et al.*, 1986; Franko *et al.*, 1987; and see accompanying manuscript, Koch *et al.*, 1995*a*). Nitroimidazole-binding techniques allow measurements of hypoxia across individual cell distances and with a multiplicity of techniques. Early studies utilised $^{14}C$-labelled misonidazole with interpretation based on autoradiographs (Chapman *et al.*, 1983). Because this method is tedious, time consuming and not readily applicable clinically, investigators have sought to develop antibody-based detection techniques against nitroimidazole compounds. Studies have been performed using antibodies, for example against CCI-103F (Raleigh *et al.*, 1987; Cline *et al.*, 1994) and 7-(4"-(2-nitroimidazole-1-yl)-butyl)-theophylline (NITP) (Hodgkiss *et al.*, 1992*a*,*b*). Examination of binding has included analysis of tissue sections stained via fluorescence (Hodgkiss *et al.*, 1991) and immunohistochemical techniques (Cline *et al.*, 1994). Flow cytometric techniques to measure binding to individual cells have also been described (Hodgkiss *et al.*, 1991; Olive and Durand, 1983). Recently, a monoclonal antibody was raised against adducts of a pentafluorinated derivative of etanidazole, [2-(2-nitro-1H-imidazol-1-yl)-*N*-(2,2,3,3,3-pentafluoropropyl) acetamide] (EF5) (Lord *et al.*, 1993) and binding in tumour cells was visualised using fluorescence immunohistochemical techniques (Koch *et al.*, 1995*a*). We have used this technique to assess hypoxia in an implanted rat glioma model.

In the mid-1960s, a glioma tumour was induced in a male CD Fischer rat following weekly injections of *N*-nitrosomethylurea. After successive *in vivo* and *in vitro* transfers, a cell line was established (9L) which produced a gliosarcoma when implanted intracerebrally. Since that time, the 9L has been used extensively as a subcutaneous and intracerebral tumour model, especially for studies of radiosensitivity (Leith *et al.*, 1975; Wallen *et al.*, 1980). Recently, this tumour has been described as a tissue isolate grown on the epigastric branch of the femoral vessels (Evans and Koch, 1994). Typically, intracerebral and small subcutaneous 9L tumours are characterised as having minimal necrosis and no severe hypoxia (Leith *et al.*, 1975; Wallen *et al.*, 1980). However the 9L glioma has also been reported to contain uniform or moderate, intermittent hypoxia (Moulder and Rockwell, 1984; Wong *et al.*, 1990; Franko *et al.*, 1992).

The purpose of this study was to utilise *in vivo* EF5 binding as detected by a fluorochrome-conjugated monoclonal antibody to demonstrate chronic tumour hypoxia. We chose to investigate these techniques in the 9L glioma tumour, in part because the exact nature of the distribution of hypoxia in this tumour system is controversial. Recent work from our laboratory has confirmed that the oxygen dependence of binding in 9L (and WNRE cells) is the same for radioactive and monoclonal antibody-based detection measurements of drug uptake (Koch *et al.*, 1995*a*). Furthermore, these studies have indicated that the relative fluorescence of cells from 9L tumours incubated with EF5 corresponded to the oxygen concentration at which they were incubated (Koch *et al.*, 1995*a*).

### Materials and methods

### *Drug synthesis, preparation of monoclonal antibodies and EF5 binding-fluorescence assay*

These aspects are described in the accompanying manuscript (Koch *et al.*, 1995*a*).

### *Cell preparation*

9L rat glioma cells (Wallen *et al.*, 1980; Franko *et al.*, 1992) were obtained from KT Wheeler (Bowman Gray School of Medicine, Winston Salem, NC, USA). Tumours were

Correspondence: SM Evans, University of Pennsylvania, School of Veterinary Medicine, 3850 Spruce St., Philadelphia, PA 19104, USA.
Received 15 December 1994; revised 2 May 1995; accepted 11 May 1995

initiated by injection of cells or tissue chunks as described previously (Evans and Koch, 1994). The dissociation of tumour cells used previously described methods (Howell and Koch, 1980; Evans and Koch, 1994) except that 10 ml of enzymatic cocktail (protease, collagenase and DNAase) was used for tissue samples ranging from 300 to 500 mg.

*Tumour tissue samples*

All animal studies were performed under the regulations of the University of Pennsylvania Institutional Animal Care and Use Committee (IACUC). 9L tumours were grown as tissue-isolated implants on the epigastric artery and vein as described previously (Evans and Koch, 1994). The rat was given EF5 as an intravenous injection of 10 mM EF5 prepared in 0.9% saline. The mass of solution administered was 1% of the rat's mass; thus the equivalent whole-body concentration was 100 μM. In mice, the whole-body distribution of EF5, determined using $^{14}$C-labelled EF5 at 0.5 h post injection was very uniform (Laughlin, 1995); similar data in rats are not available currently. Three hours following EF5 administration, anaesthesia was induced with xylazine (1.3 mg $kg^{-1}$ i.p.) and ketamine (140 mg $kg^{-1}$ i.p.), the tumour removed and immediately cooled. The serum half-life of EF5 in rats is about 150 min, so rapid cooling is necessary to prevent depletion of oxygen followed by binding of residual drug in the excised tissue (Koch *et al.*, 1993). The tumour was weighed and then bisected. Half of the tumour was used for disaggregation (Howell and Koch, 1980; Evans and Koch, 1994) and cell analysis (plating efficiency, analysis of EF5 binding by flow cytometry) and the other half was quickly frozen for histopathological analysis. The tissue was placed onto a small piece of saline-moistened filter paper, frozen in ethanol or isopentane at − 50°C, solvent was rinsed off by immersion for a few seconds in brine at − 15°C, brine rinsed off by ice-water slush, and then the still frozen tissue specimen placed onto solid carbon dioxide pellets. Frozen tissue was stored in small, closed containers at − 80°C until sectioning.

Tumour sections were cut at 14 μm thickness using a Microm HM 505 N cryostat and collected onto poly-L-lysine-coated microscope slides. Staining of the tissue sections was the same as previously described for whole cells in the accompanying manuscript (Koch *et al.*, 1995*a*), except that rinses were done by moving each tissue section from container to container. Note that residual unbound drug is removed immediately during the fixation stage. Tissue sections were photographed using a Nikon fluorescence microscope, with a tetramethylrhodamine filter set. A 10 × Fluor objective allowed typical exposure times of 6–400 s with the 100 W high-pressure mercury light source. An infrared cut-off filter was used for both photography (Kodak Ektachrome 'Elite 400' slide film) and photometry. Photometric analyses were performed by centring the microscopic field on an appropriate area of the tissue section being analysed and then noting the number of seconds that would be necessary to appropriately expose the Kodak Ektachrome 'Elite 400' film (Nikon UFX-IIA; large focal spot). These numbers were then used as a means for comparison of fluorescence intensity among tumour regions ('photometry'). Photographic slide images were digitised with a Nikon 'Cool-Scan' and analysed using the NIH 'Image' software and Adobe Photoshop.

We have found that the fluorescent signal is most stable if the sections are kept in cold phosphate-buffered saline (PBS)/1% paraformaldehyde. Since we wanted to be able to photograph both fluorescence and conventional staining of the same section, a special coverslip system was devised. This consisted of two strips of mylar film attached to the slide with glycerol, covered with a haemocytometer coverslip. The resulting capillary space was filled with PBS/1% paraformaldehyde and kept at 4°C and 100% humidity (a conventional coverslip is not strong enough to resist the effects of moderate desiccation within the capillary space during photography at room temperature). Photography of antibody fluorescence was made at noted vernier locations on the tissue section. The coverslip was carefully removed after immersion of the slide in a jar of PBS. The slide was then removed, air dried and stained with haematoxylin and eosin (H&E), followed by relocation of the original vernier settings and conventional photography.

*Plating efficiency*

For clonogenic assay, suitable numbers of cells were plated into 100 mm plastic Petri dishes. Each dish contained 9 ml of Eagle's minimal essential medium (MEM) made with 13% (v/v) bovine calf serum and 1% antibiotics (Gibco), 'complete medium' and 50 000 feeder cells (feeder cells were prepared by irradiating 9L cells from tissue culture with 25 Gy). The number of cells plated was varied over a range in order to yield 100–200 colonies per plate. In this range, the number of colonies varies linearly with the number of 9L cells seeded. Multiple replicates were plated at each of 3–5 dilutions. The plates were incubated for 10–12 days followed by fixation, staining and counting of colonies.

*Irradiation studies*

Radiation was performed on an orthovoltage X-ray unit operated at 225 kVp and 10 mA, 0.2 mm copper filter. The dose rate was 4.0 Gy $min^{-1}$. Doses were estimated based on actual surface dosimetry for each tumour using thermoluminescent devices. Hypoxia was induced in tumours by allowing 10 min following euthanasia before irradiation. Tumours were irradiated with doses of 0–30 Gy.

### Results

Figure 1 illustrates the radiation response of 9L epigastric implants following 0–30 Gy radiation in air-breathing *vs* euthanised rats. Also shown is the radiation response of cells dissociated from 9L tumours and irradiated as a cell suspension in room air. In our model, the oxygen enhancement ratio for a surviving fraction of 1% is 2.9. The radioresponse of our epigastric tumours in euthanised rats was similar to that reported by Wallen *et al.* (1980), when corrected for our slightly higher plating efficiency: 22% *vs* 15%. However, the surviving fraction of tumours irradiated in air-breathing rats

**Figure 1** Demonstrates the plating efficiency of cells from epigastric 9L implants following 0–30 Gy radiation in air-breathing compared with euthanised rats. 9L tumor cells irradiated in suspension are included for comparison. ■, euthanised; ●, air-breathing; Δ, tumour cells irradiated in suspension. Small dots and solid lines represent data from unweighted quadratic fit. For euthanised rats, $PE_{0\,Gy} = 0.24$, alpha = 0.039 $Gy^{-1}$ and beta = 0.003 $Gy^{-2}$. For air-breathing rats, $PE_{0\,Gy} = 0.17$, alpha = 0.142 $Gy^{-1}$ and beta = 0.001 $Gy^{-2}$. For cells irradiated *in vitro*, $PE_{0\,Gy} = 0.26$, alpha = 0.205 $Gy^{-1}$ and beta = 0.002 $Gy^{-2}$.

is substantially higher than that reported by Wallen *et al.*, (1980). As discussed below, the presence of significant hypoxia based on EF5 binding is consistent with this finding.

The distribution of fluorescent binding of EF5 as a measure of hypoxic heterogeneity in 9L epigastric tumour tissue sections was studied. Two 9L tumours, representing opposite ends of the spectrum of EF5 binding are illustrated. Tumour A was generally characterised by the presence of significant binding of EF5 (Figure 2) compared with tumour B (Figure 3) which had a few areas of EF5 binding but was generally dim. Evaluation of these tumour specimens was based upon overall fluorescence intensity patterns of distribu-

**Figure 2** Representative fields from tumour A tissue sections. Top (**a**–**d**): Tumour was excised 3 h following EF5 administration and immediately frozen. Fourteen micron tumour sections were stained with Cy-3-conjugated ELK3-51 and photographed with epifluorescent illumination using a rhodamine filter set. A 10 × Fluor objective allowed an exposure time of 90 s using a 100 W light source without attenuation. Kodak Ektachrome 'Elite 400' slide film was used. Following photography of the 1050 × 700 μm regions, the tumour sections were stained with haematoxylin and eosin and the same sections were rephotographed. (**a**) 5 s exposure of a region of widely varying intensity, consistent with the 'Thomlinson and Gray' pattern of binding. (**b**) 5 s exposure of a region characterised by uniformly intense binding. (**c**) 5 s exposure of a region characterised by minimal binding. (**d**) 84 s exposure of same area as seen in (**c**) showing that even in relatively oxic regions, variations in binding can be demonstrated. Bottom (**e**–**g**) Haematoxylin and eosin staining of corresponding regions as described for fluorescence photography above. (**h**) NIH 'Image' topographical representation of same field as (**a**).

**Figure 3** (**a**) 90 s exposure of 14 μm frozen section of 1050 × 700 μm areas of 9L epigastric tumour B as viewed under the fluorescent microscope. Rat was treated and tissues prepared as described in Figure 2. (**b**) Corresponding section stained with haematoxylin and eosin demonstrating a triangular region of hypoxia surrounding necrosis, despite the presence of apparently oxic tumour in close proximity.

tion. In tumour A the fluorescence distribution patterns are of two types. In the first type, the intensity varies substantially over several hundred microns of tissue (Figure 2a). This pattern is characteristic of the 'Thomlinson and Gray' distribution (Gray *et al*., 1953; Thomlinson and Gray, 1955) with variations of fluorescent staining occurring over 100–250 μm distances, corresponding to known oxygen diffusion ranges in tumour tissue. The junctional areas between high and low binding show changes in fluorescence from maximal to minimal binding over small distances (< 100 μm). The second pattern seen in tumour A is characterised by moderately to fully hypoxic regions over larger distances (>> 300 μm). The fluorescence distribution in these regions have larger areas of relatively homogeneous binding (Figure 2b). On H&E sections, this tumour region is relatively homogeneous with minimal evidence of necrosis (Figure 2f). Areas of tumour necrosis are relatively uncommon in the 9L tumours we have studied. However, we have seen regions of necrosis in some 9L epigastric tumours which have minimal EF5 binding. Tumour B, illustrated in Figure 3, is characterised by large regions without EF5 binding, apparently oxic tumour cells. Figure 3 also illustrates that low levels or the absence of binding is related to two separate and distinct processes. The first is the presence of viable, oxic cells which do not bind EF5 as described above. The second is related to regions of cell death where the cells are hypoxic but are not metabolically able to reduce and bind the EF5. Also of interest in tumour B is the presence of many pyknotic cells in the region of high binding (Figure 3). Based upon the number and distribution of these cells, at least some of them are still able to metabolise EF5.

Figure 4 demonstrates the distribution of fluorescent cells from the same two 9L epigastric tumours, A and B, as determined by flow cytometric analysis of fluorescent antibodies specific for EF5 binding. Based upon the dot-plot distributions (forward *vs* side scatter), three separate cellular subpopulations (R1–3) can be identified in both tumours. Despite the presence of each population in both tumours, the relative numbers of each cellular subgroup varies. Tumour A has many more cells in the R1 region. Previous data from this laboratory (see Koch *et al.*, 1995*a*) has shown that there is little EF5 binding to the R1 cells; the exact nature of these cells (or portions of cells) has not been determined, but they do not metabolise EF5 when incubated in nitrogen *in vitro*. Population R2 is characterised by cells that are relatively small in size and complexity, with a median fluorescence of approximately 15; these cells most likely represent lymphocytes. The remaining cells, R3, are generally larger (higher forward scatter) and have variable complexity (side scatter) compared with population R1 or R2. In tumour A, R3 contains two distinct cell subpopulations with fluorescent peaks between 10 and 1000. The average relative fluorescence of the brightest cells in R3 of either tumour is 500–600 compared with the average relative fluorescence of the cells in R2, which are approximately 10–30 (a contrast between the most and least hypoxic cells of approximately 16–60 ×). In addition, the most hypoxic R3 cells (greater than $10^3$ relative fluorescence) are absent in tumour B; this corresponds well to the overall appearance of this tumour on fluorescent microscopy which demonstrated only a few regions of moderate EF5 binding.

Another method for quantification of the overall fluorescence intensity, and therefore relative hypoxia, in each section is to compare the photometry reading in various

**Figure 4** The dot-plot distributions (right) and flow cytometric analyses (left) of cells dissociated from 9L epigastric tumours **A** and **B** three hours following EF5 administration, at which time the tumour was excised and dissociated into individual cells. The upper panels represent data from the tumour shown in Figure 2 and the lower panels represent data from the tumour shown in Figure 3. Based upon the dot-plot distributions, three different cell subpopulations (R1–3) can be identified. Population R1 is believed to comprise red blood cells, platelets and tumour debris, explaining minimal EF5 binding. Population R2 is characterised by relatively small size and complexity, has low fluorescence and most likely represents lymphocytes. Population R3 is generally larger in size (higher forward scatter) and has variable complexity (side scatter) compared with populations R1 or R2. In tumour A shown in the upper panel, R3 contains two distinct cell subpopulations with fluorescent peaks between $10^2$ and $10^3$. For tumour B, lower panel, there are very few cells in the second R3 peak.

regions of the tumour. In tumour A, the brightest regions had a photometry reading of 5 s compared with the dimmest regions where the reading was 84 s. This 17-fold intratumoral contrast factor within a single tumour section is within the range of contrast between the brightest and dimmest cells in the entire tumour, based upon flow cytometric analyses (16–60×).

### Discussion

The 9L glioma has been used extensively as a model for studies of radiotherapy (Leith *et al.*, 1975; Wallen *et al.*, 1980). Our data support the resistant nature of the 9L glioma shown by previous investigators (Leith *et al.*, 1975; Wallen *et al.*, 1980). The large difference in radiation response between tumours irradiated under air-breathing conditions *in vivo vs* cells from tumours irradiated in suspension under aerobic conditions suggests the influence of a contact effect and/or the effect of hypoxia. However, it is unlikely that the contact effect can explain these results because aerobic cells from tumours and aerobic cells in tissue culture have the same radiation response (data not shown). The exact nature of hypoxia in the 9L has remained elusive. Evidence for radioresistant hypoxic cells was not found in 9L spheroids which contained necrotic centres (Gutin *et al.*, 1982). However, carbogen breathing combined with Fluosol-DA was shown to sensitise intracranial 9L tumours to radiation (Teicher *et al.*, 1988). In 1992, Franko *et al.* sought a more direct analysis of oxygen concentration using autoradiograms of $^3$H-misonidazole ($^3$H-MISO)-labelled 9L tumours and spheroids. In spheroids, the binding of [$^3$H]MISO varied inversely to oxygen concentration. Cells adjacent to the necrotic centre bound [$^3$H]MISO, but these cells were found to be non-clonogenic. In 9L tumours labelled *in vivo*, the labelling rose gradually from the periphery of the tumour to the centre and cells adjacent to the rare necrotic areas appeared to be severely hypoxic.

In 0.05 g intracranial tumours, less than 0.35% (Wallen *et al.*, 1980) and 0.6–2.6% (Leith *et al.*, 1975) hypoxic cells were reported. In subcutaneous 9L tumours, hypoxic fractions of 0.9–13% are reported (Wallen *et al.*, 1980). These data were based upon analysis of non-parallel paired survival curves. As described by Moulder and Rockwell (1984), such analysis requires several assumptions and therefore, the hypoxic fraction may not be determined unambiguously. None the less, these data have been described as being compatible with a compartment of 3.1% fully radioresistant, hypoxic cells *or* a larger fraction of moderately radioresistant cells *or* a mix of moderately and fully hypoxic cells. The data on EF5 binding presented herein may shed some light on this question. Two overall patterns of EF5 binding have been found in 9L epigastric tumours. One type corresponds well to the patterns expected based upon diffusion and metabolism of oxygen from individual vessels, 'chronic hypoxia', and described in the 1950s by Thomlinson and Gray (Gray *et al.*, 1953; Thomlinson and Gray, 1955). The second pattern is characterised by moderate hypoxia over several millimetre distances. These regions are more difficult to explain physiologically than the 'Thomlinson and Gray' pattern, but several hypotheses are suggested: episodes of acute hypoxia (Chaplin *et al.*, 1986); regions of cells with low(er) oxygen consumption and/or a combination of the capillary distribution and tumour oxygen consumption. The last situation has been predicted by Secomb *et al.* (1993) under conditions of moderate oxygen consumption ($0.23\,cm^3\,O_2$ per 100 gm $min^{-1}$) and relatively low capillary density, wherein regions of $pO_2$ less than 1 mmHg are likely. Both the distribution and absolute brightness of fluorescence seen in our antibody-stained sections and flow cytometric analysis of cells and tissues, respectively, suggest extensive hypoxia in the 9L tumours we have been studying. This is entirely consistent with the high degree of radiation resistance seen in our lines irradiated *in situ* in air-breathing *vs* euthanised rats. Our data is internally consistent with a large proportion of moderate to fully hypoxic cells in these relatively more radioresistant tumours; we still do not have a complete explanation for the various theories concerning the degree of hypoxia in 9L tumours. We know, however, that the binding patterns described herein are not restricted to the use of the epigastric model because the same degree of binding and radioresistance has been found in the subcutaneously implanted 9L tumour model (Evans and Koch, 1994; and work in progress). It is possible that the average level of hypoxia, or its distribution within a tumour varies substantially within various laboratories. One example of both inter- and intratumour heterogeneity is the interesting pattern of binding seen in tumour B, Figure 3. In this small triangular region, with only moderate levels of binding, we find necrosis. Yet, in tumour A (Figure 2) much larger regions with undoubtedly much lower oxygen levels remain viable. Clearly, there are interesting interplays of nutrient presentation and utilisation throughout these tumours that are not yet completely understood. Further studies emphasising the relative location of vasculature *vs* proliferating and quiescent cells with hypoxia are under way.

One of the most interesting but troublesome problems in tumour biology and therapy is the presence of inter- and intratumoral heterogeneity. Variations in cellular oxygenation (as a result of tumour blood flow) is one of the most important heterogeneous parameters of tumours because this characteristic affects pH, nutritional and growth factors, accumulation of cellular waste products, and energy sources. The ability to carefully examine and compare the distribution of cellular oxygenation within individual or in different tumours is provided by the EF5-binding technique because it demonstrates variation in binding over cell–cell distances. For a quick 'snapshot' of the average and distribution of cellular oxygenation, flow cytometry can be used. In the two tumours presented here, significant inter- and intratumour variability in oxygen levels are demonstrated. As well, unique information on the presence of multiple cellular populations can be inferred. Both the flow cytometric and fluorescent immunohistochemical techniques can be extended to investigate the relationship between hypoxia and other parameters such as proliferation (Zeman *et al.*, 1993), host cell distribution (MacDonald and Koch, 1977; Nathan *et al.*, 1982; Loeffler *et al.*, 1990), vascular distribution (Weber *et al.*, 1985), apoptosis (Muschel *et al.*, 1995), activation of cellular regulation factors (for review see Brown and Giacci, 1994) and hyperthermia (Koch *et al.*, 1995*b*; Oleson, 1995).

Flow cytometric analysis of EF5 binding provides an evaluation of the various cell populations and fluorescent distribution of cell types within the tumour, as well as the percentage of maximally hypoxic cells. It is of interest that in both the 9L epigastric tumours shown, three cell populations (based upon cell size and complexity) were seen. The first population (R1) bound little, if any, EF5 and most likely represents cellular debris, red blood cells, platelets, etc. The second population (R2) is also relatively homogeneous in size, complexity and EF5 binding. This most likely represents lymphocytes, based upon known discrimination of normal blood cell populations using light scattering (size, detected by forward angle light scattering and granularity, assessed by side light scattering; Thompson *et al.*, 1985). The third population of cells are of relatively similar size and complexity but yet are clearly separate populations when analysed for fluorescence. At this time it is unknown whether these are two distinct tumour cell populations or whether one of them represents host cells, such as monocytes or macrophages. Monoclonal antibody-based studies against rat haematopoietic cell surface markers are currently under way. Other explanations include technical considerations such as the possibilities of doublets or biological effects such as cell cycle and metabolism.

One of the many interesting questions that can be answered using this technique is the fate of hypoxic tumour cells. Indirect measures of 9L tumour hypoxia give the impression that the 9L tumour contains few hypoxic cells. However, cells adjacent to the necrotic centre of 9L spheroids bound [$^3$H]MISO; in spheroid 'cure experiments' these cells were not found to be clonogenic (Franko *et al.*, 1992). Conversely, however, the hypoxia identified by the EF5-binding technique is likely to account for the radiation resistance of the 9L epigastric tumours studied herein. Additional studies comparing EF5 binding and tumour growth delay would be necessary to further evaluate this observation. Studies on the relationship between the presence, distribution and number of hypoxic cells and their role in tumour persistence are currently ongoing. The results of such studies would be expected to vary between tumour types and for individual tumours within a given type. It is this type of information that is critical for the evaluation of individual human tumours in order to predict therapeutic tumour response.

Photomicrographs provide specific information on the distribution of hypoxic cells and the tumour's overall heterogeneity. Our continuing studies are aimed at determining whether the overall level of hypoxia, as predicted from the flow cytometric data, correlates with the number, level, and distribution of hypoxic cells in photomicrographs. It is not known at this time whether the tumour's average level of hypoxia, the number of maximally hypoxic cells or the heterogeneity of these characteristics determine the therapeutic response of a given tumour. Indeed, as noted above, in some tumours the presence of hypoxic cells may not be the factor which limits survival. As demonstrated herein, the excellent fluorescent contrast provided by EF5 binding with monoclonal antibody detection will allow the analysis of these questions.

**Acknowledgements**

Work supported by grants CA-56679 (SME, CJK) and CA-28332 (EML) from the National Institutes of Health and the Department of Radiation Oncology, University of Pennsylvania.

### References

BROWN JM AND GIACCI AJ. (1994). Tumor hypoxia: the picture has changed in the 1990's. *Int. J. of Radiation Biology*, **65**, 95–102.

CHAPLIN DJ, DURAND RE AND OLIVE PL. (1986). Acute hypoxia in tumors: Implications for modifiers of radiation effects. *Int. J. Rad. Oncol. Biol. Phys.*, **12**, 1279–1282.

CHAPMAN JD, BAER K AND LEE J. (1983). Characteristics of the metabolism-induced binding of misonidazole to hypoxic mammalian cells. *Cancer Res.*, **43**, 1523–1528.

CLINE JM, THRALL DE, ROSNER GL AND RALEIGH JA. (1994). Distribution of the hypoxia marker CCI-103F in canine tumors. *Int. J. Radiat. Oncol. Biol. Phys.*, **28**, 921–933.

EVANS SM AND KOCH CJ. (1994). Characterization of the 9L glioma as a tissue isolated epigastric implant. *Radiat. Oncol. Invest.*, **2**, 134–143.

FRANKO AJ, KOCH CJ, GARRECHT BM, SHARPLIN J AND HUGHES D. (1987). Oxygen dependence of binding of misonidazole to rodent and human tumors in vitro. *Cancer Res.*, **47**, 5367–5376.

FRANKO AJ, KOCH CJ AND BOISVERT DPJ. (1992). Distribution of misonidazole adducts in 9L gliosarcoma tumors and spheroids: Implications for oxygen distribution. *Cancer Res.*, **52**, 1–7.

GRAY LH, CONGER AD, EBERT M, HORNSEY S AND SCOTT OCA. (1953). Concentration of oxygen dissolved in tissues at the time of irradiation as a factor in radiotherapy. *Br. J. Radiol.*, **26**, 638–648.

GUTIN PH, BARCELLOS MH, SHRIEVE DC, SANO Y, BERNSTEIN M AND DEEN DF. (1982). Further evidence for the absence of a hypoxic fraction in the 9L rat tumor muticell spheroid system. *Br. J. Cancer*, **55**, 688–690.

HODGKISS RJ, JONES G, LONG A, PARRICK J, SMITH KA, STRATFORD MRL AND WILSON GD. (1991). Flow cytometric evaluation of hypoxic cells in solid experimental tumours using fluorescence immunodetection. *Br. J. Cancer*, **63**, 119–125.

HODGKISS RJ, MIDDLETON RW, PARRICK J, RAMI HK, WARDMAN P AND WILSON GD. (1992*a*). Bioreductive fluorescent markers for hypoxic cells: A study of 2-nitroimidazoles with 1-substituents containing fluorescent, bridgehead-nitrogen, bicyclic systems. *J. Med. Chem.*, **35**, 1920–1926.

HODGKISS RJ, KELLEHER E AND PARRICK J. (1992*b*). Hypoxia-specific inhibition of recovery from radiation damage by a novel 2-nitroimidazole with a theophylline side chain. *Int. J. Radiat. Biol.*, **61**, 797–804.

HOWELL RL AND KOCH CJ. (1980). The disaggregation, separation and identification of cells from irradiated and unirradiated EMT6 mouse tumors. *Int. J. Radiat. Oncol. Biol. Phys.*, **6**, 311–318.

KOCH CJ, GIANDOMENICO AR AND LEE IYENGAR CW. (1993). Bioreductive metabolism of AF-2 [2(2-furyl)-3-(5-nitro-2-furyl)-acrylamide] combined with 2-nitroimidazole radiosensitizing agents. *Biochem. Pharmacol.*, **46**, 1029–1036.

KOCH CJ, EVANS SM AND LORD EM. (1995*a*). Oxygen dependence of cellular uptake of EF5 [2-(2-nitro-1H-imidazol-1-yl)-N-(2,2,-3,3,3-pentafluoropropyl) acetamide]: Analysis of drug adducts by fluorescent antibodies *vs* bound radioactivity. *Br. J. Cancer*, **72**, 865–870.

KOCH CJ, EVANS SM AND LORD EM. (1995*b*). Comment on the hypothesis that hyperthermia facilitates reoxygenation. *Int. J. Hyperthermia*, **2**, 447–450.

LAUGHLIN KM, EVANS SM, LORD EM AND KOCH CJ. (1995). Biodistribution of the nitroimidazole EF5 (2-[2-nitro-1h-imidazol-1-yl]-n-(2,2,3,3,3-pentafluoropropyl) acetamide) in mice bearing subcutaneous EMT6 tumors. *Journal of Pharmacology and Experimental Therapeutics*. (submitted).

LEITH JT, SCHILLING WA AND WHEELER KT. (1975). Cellular radiosensitivity of a rat brain tumor. *Cancer*, **35**, 1545–1550.

LOEFFLER DA, KENG PC, BAGGS RB AND LORD EM. (1990). Lymphocyte infiltration and cytotoxicity under hypoxic conditions in the EMT6 mouse mammary tumor. *Int. J. Cancer*, **45**, 462–467.

LORD EM, HARWELL L AND KOCH CJ. (1993). Detection of hypoxic cells by monoclonal antibody recognizing 2-nitroimidazole adducts. *Cancer Res.*, **53**, 5271–5276.

MACDONALD HR AND KOCH CJ. (1977). Energy metabolism and T cell mediated cytolysis I. Synergism between inhibitors of respiration and glycolysis. *J. Exp. Med.*, **146**, 698–709.

MOULDER JE AND ROCKWELL SC. (1984). Hypoxic fractions of solid tumors: experimental techniques, methods of analysis and a survey of existing data. *Int. J. Radiat. Oncol. Biol. Phys.*, **10**, 695–712.

MUSCHEL RJ, BERNHARD E, GARZA L, MCKENNA WG AND KOCH CJ. (1995). Induction of apoptosis under hypoxic conditions. *Cancer Res.*, **55**, 995–998.

NATHAN CF, MERCER-SMITH JA, DESANTIS NM AND PALLADINO MA. (1982). Role of oxygen in T cell mediated cytolysis. *J. Immunol.*, **129**, 2164–2171.

OLESON JR. (1995). Hyperthermia from the clinic to the laboratory: An Hypothesis. *Int. J. Hyperthermia*, **2**, 315–322.

OLIVE PL AND DURAND RE. (1983). Fluorescent nitroheterocycles for identifying hypoxic cells. *Cancer Res.*, **43**, 3276–3280.

RALEIGH JA, MILLER GG, FRANKO AJ, KOCH CJ. FUCIARELLI AF AND KELLEY DA. (1987). Fluorescence immunohistochemical detection of hypoxic cells in spheroids and tumours. *Br. J. Cancer*, **56**, 395–400.

SECOMB TW, HSU R, DEWHIRST MW, KLITZMAN B AND GROSS JF. (1993). Analysis of oxygen transport to tumor tissue by microvascular networks. *Int. J. Radiat. Oncol. Biol. Phys.*, **25**, 481–489.

STONE HB, BROWN MJ, PHILLIPS TL AND SUTHERLAND RM. (1993). Oxygen in human tumors: correlations between methods of measurement and response to therapy. *Radiat. Res.*, **136**, 422–434.

TEICHER BA, HERMAN TS AND ROSE CM. (1988). Effect of Fluosol-DA on the response of intracranial 9L tumors to X-rays and BCNU. *Int. J. Radiat. Oncol. Biol. Phys.*, **15**, 1187–1192.

THOMLINSON RH AND GRAY LH. (1955). The histological structure of some human lung cancers and the possible implications for radiotherapy. *Br. J. Cancer*, **9**, 539–579.

THOMPSON JM, GRALOW JR, LEVY R AND MILLER RA. (1985). The optimal application of forward and ninety degree light scatter in flow cytometry for the gating of mononuclear cells. *Cytometry*, **6**, 401–406.

URTASUN RC, CHAPMAN JD, RALEIGH JA, FRANKO AJ AND KOCH CJ. (1986). Binding of 3H-Misonidazole to solid human tumors as a measure of tumor hypoxia. *Int. J. Radiat. Oncol. Biol. Phys.*, **12**, 1263–1267.

WALLEN CA, MICHAELSON SM AND WHEELER KT. (1980). Evidence for an unconventional radiosensitivity of rat 9L subcutaneous tumors. *Radiat. Res.*, **85**, 529–541.

WEBER T, SEITZ RJ, LIEBERT UG, GALLISH E AND WECHSLER W. (1985). Affinity cytochemistry of vascular endothelia in brain tumors by biotinylated Ulex Europaeus Type I Lectin (UEA I). *Acta Neuropathol. (Berl)*, **67**, 128–135.

WONG KH, WALLEN CA AND WHEELER KT. (1990). Chemosensitization of the nitrosoureas by 2-nitroimidazoles in the subcutaneous 9L tumor model: Pharmacokinetic and structure activity considerations. *Int. J. Radiat. Biol. Oncol. Phys.*, **18**, 1043–1050.

ZEMAN EM, CALKINS DP, CLINE JM, THRALL DE AND RALEIGH JA. (1993). The relationship between proliferative and oxygenation status in spontaneous canine tumors. *Int. J. Radiat. Oncol. Biol. Phys.*, **27**, 891–898.